# Monocytic Cell Adhesion to Oxidised Ligands: Relevance to Cardiovascular Disease

**DOI:** 10.3390/biomedicines10123083

**Published:** 2022-11-30

**Authors:** Robin N. Poston, Jenna Chughtai, Desara Ujkaj, Huguette Louis, David S. Leake, Dianne Cooper

**Affiliations:** 1 Centre for Microvascular Research, William Harvey Research Institute, Faculty of Medicine and Dentistry, Queen Mary University of London, Charterhouse Square, London EC1M 6BQ, UK; 2 INSERM U1116, 54505 Vandoeuvre-Les-Nancy, France; 3 School of Biological Sciences, Institute of Cardiovascular and Metabolic Research, University of Reading, Reading RG6 6EX, UK; 4 Centre for Biochemical Pharmacology, William Harvey Research Institute, Faculty of Medicine and Dentistry, Queen Mary University of London, Charterhouse Square, London EC1M 6BQ, UK

**Keywords:** monocyte, adhesion, CD14, TLR4, raft, oxidation, LDL, phospholipid, atherosclerosis, thrombosis

## Abstract

Atherosclerosis, the major cause of vascular disease, is an inflammatory process driven by entry of blood monocytes into the arterial wall. LDL normally enters the wall, and stimulates monocyte adhesion by forming oxidation products such as oxidised phospholipids (oxPLs) and malondialdehyde. Adhesion molecules that bind monocytes to the wall permit traffic of these cells. CD14 is a monocyte surface receptor, a cofactor with TLR4 forming a complex that binds oxidised phospholipids and induces inflammatory changes in the cells, but data have been limited for monocyte adhesion. Here, we show that under static conditions, CD14 and TLR4 are implicated in adhesion of monocytes to solid phase oxidised LDL (oxLDL), and also that oxPL and malondialdehyde (MDA) adducts are involved in adhesion to oxLDL. Similarly, monocytes bound to heat shock protein 60 (HSP60), but this could be through contaminating lipopolysaccharide. Immunohistochemistry on atherosclerotic human arteries demonstrated increased endothelial MDA adducts and HSP60, but endothelial oxPL was not detected. We propose that monocytes could bind to MDA in endothelial cells, inducing atherosclerosis. Monocytes and platelets synergized in binding to oxLDL, forming aggregates; if this occurs at the arterial surface, they could precipitate thrombosis. These interactions could be targeted by cyclodextrins and oxidised phospholipid analogues for therapy.

## 1. Introduction

Atherosclerosis is a focal chronic inflammatory process of arteries that is a major cause of morbidity and mortality throughout the world [1]. It primarily involves the intimal layer of the arterial wall, into which monocytes and lymphocytes traffic from the blood. This traffic is likely to be a critical and rate limiting factor in the disease [2], and is shown by monocyte traffic and accumulation correlating strongly with the existence of the focal lesions of the disease, with little seen away from them. It is thought that the initiating factor is the accumulation of LDL in the arterial intima: LDL was recently found to be transported across the luminal endothelial layer by a specific scavenger receptor B1 dependent mechanism [3,4]. LDL is an unstable micelle, in which the surface phospholipid oxidizes readily in vitro, giving rise to the initial product, minimally modified LDL (mmLDL), which can be followed by further oxidation of the apolipoprotein B100 protein to give fully oxidised LDL (oxLDL). Minimally modified LDL contains oxidised phospholipids [5] that can be recognized by EO6 antibody [6], while in fully oxidised LDL the apolipoprotein B (apoB) is modified by malondialdehyde (MDA) adduct formation, detectable by MDA2 antibody [7].

The generally accepted mechanism for plaque development is that ox-LDL or related products found in the arterial intima [8,9,10] are initially responsible for the induction of adhesion molecules that are found on the luminal endothelium of atherosclerotic plaques [11,12,13]. These can then bind monocytes and initiate traffic, leading to the first stages of the lesions. Both endothelial cells and macrophages can themselves amplify the process by oxidizing LDL [14,15]. Further, macrophages, derived from the monocyte traffic, accumulate in the developing plaques, and are also activated by modified LDL present in the arterial intima [16,17,18]. They secrete cytokines which activate the luminal endothelium, to express adhesion molecules such as P-selectin and ICAM-1 [19] together with chemokines such as CCL2 (MCP-1) that directly attract monocytes [20]. In this manner, the macrophage accumulation in the plaque is likely to become a self-perpetuating process, a mechanism important in focal plaque generation [2]. Nevertheless, multiple attempts at the therapeutic use of agents against the range of adhesion molecules implicated to date have been unsuccessful [21].

In this study, we show a novel additional action for oxLDL, that it can directly induce monocyte adhesion via a CD14 and Toll-like receptor 4 (TLR4) dependent mechanism, and propose that adhesion to oxLDL or its components may be a contributory factor to plaque generation and thrombosis. CD14 is a glycophosphatidylinositol (GPI) linked cell surface molecule, highly expressed on monocytes and macrophages, that acts as a co-receptor with the TLR2 and TLR4 [22]. This receptor complex can exist in localised cholesterol rich membrane microdomains, known as lipid rafts, particularly after binding its ligands, such as bacterial lipopolysaccharide (LPS) [23]. The lipid rafts can be disrupted by cholesterol depletion with methyl β cyclodextrin (MCD) [24] and nystatin [24]. Also the lipid solvent dimethyl sulfoxide (DMSO) can modify their structure [25,26]. The TLRs in the complex induce a cascade of inflammatory signalling on ligation [27]. Importantly, the CD14-TLR4 receptor complex interaction with oxLDL also induces cytokine release from macrophages and endothelial cells through binding to endogenous oxidised phospholipids [18].

The first information suggesting a role for CD14 in monocyte adhesion was provided by Beekhuizen et al. (1991) [28], who found that a CD14 antibody inhibited the adhesion of monocytes to cultured endothelial cells. Subsequently Poston and Johnson-Tidey (1996) [29] observed that a similar antibody would strongly inhibit the binding of monocytes to both the endothelial layer and the intima of tissue sections of human atherosclerotic plaques, indicating that CD14 had a significant role in the adhesion. As ox-LDL is known to be present in atherosclerotic plaques [30], we decided to investigate whether solid phase ox-LDL could mediate monocyte adhesion. This would be a novel mechanism of monocyte adhesion to the arterial wall in atherosclerosis. The identification and understanding of this mechanism of monocyte adhesion to the arterial wall could aid the development of novel therapies for atherosclerosis.

Further, we investigated whether other ligands of CD14 and TLR4 might exist in the endothelium of human atherosclerotic arteries. It is known that the atherosclerotic endothelium suffers from oxidative stress [31], a process potentially able to generate ligands, such as oxidised phospholipids from endogenous lipids. Another stress-related potential ligand is heat shock protein 60 (HSP60), which has been reported to bind CD14 and TLR4 [32,33], but unlike other HSPs, can be present at the cell surface [34,35]. HSP60 has been shown to be present on stressed endothelial cells [34], and to be detectable on the endothelial cells of human atherosclerotic plaques [36,37]. We therefore investigated by immunohistochemistry (IHC) the presence of oxidised epitopes in these cells, and determined whether monocytes would bind to solid phase human HSP60. As with ox-LDL, if a role for HSP60 in the adhesion of monocytes to the arterial wall can be identified, it could provide a target for therapeutic intervention. However, a technical problem in investigation has been identified, as commercial sources produce recombinant HSP60 in bacteria [38]. Despite efforts to minimize contamination, bacterial lipopolysaccharide (LPS) can remain. At the time HSP60 was bought for this study, contamination could not be excluded. Interestingly, LDL shares with HSP60 the ability to bind LPS [39], and likewise the presence of LPS in LDL isolated in the laboratory is difficult to exclude. However, LDL also binds LPS in vivo, indeed it has a physiological role in transporting it to the liver after leakage from the gut, a process that leads its degradation [40]. As a consequence, the effects of bound LPS on either oxLDL or HSP60 results have to be considered in this study.

## 2. Materials and Methods

### 2.1. Human Tissues and Ethical Committee Statement

Aortas and coronary arteries were obtained from autopsies with the written permission of relatives. The autopsies were performed on hospital patients aged 60 years or more that had died from the complications of atherosclerosis or unrelated causes. Ethical permission for the research was obtained from London City and East Research Committee, ref 08/H0704/140, 30 April 2009. The study conducted according to the guidelines of the Declaration of Helsinki.

### 2.2. U937 Cell Culture

U937 cells are pro-monocytes of the human myeloid leukaemia cell lineage that mature and differentiate into monocyte-like cells upon phorbol myristate acetate (PMA) stimulation. U937 cells were obtained from the EACC, Porton Down, UK and initially grown from the frozen aliquot in 10 mL of medium. Subsequently they were maintained in suspension culture by dilution and regrowth weekly or biweekly. The culture media was comprised of RPMI 1640, 10% fetal calf serum (FCS), penicillin, streptomycin and glutamine, and was also used in the experiments.

Prior to experiments, U937 cells were stimulated with PMA 10 ng/mL, (Merck Life Science, Gillingham, UK) for 48 h. Upon stimulation, PMA activates intracellular signalling via protein kinase C and increases differentiation, with CD14 expression [41] and increased solid phase adherence. On the day of the experiment, cells were viewed under an optical microscope to confirm PMA-mediated activation, resulting in the cells becoming adherent and forming a few small visible clumps. The cells were washed and resuspended for use in the assays.

### 2.3. Monocyte Isolation

Monocytes, isolated from whole blood, were also used in adhesion assays, to assess whether data obtained from U937 cells aligned with that of human blood monocytes. Citrated whole blood, from the London Transfusion Service, was used to obtain monocytes, allowing extended time between blood withdrawal and experiments. One millilitre of 6% *w*/*v* Dextran 500 (Merck: D-1037) was added per 10 mL of blood, using 60 mL in total. The tubes were left for one hour at a 45° angle, allowing for red blood cell sedimentation. The leukocyte-rich plasma was prudently removed and placed over a NycoPrep 1.068 (Accurate Chemical, NY, USA) gradient: 3 mL of NycoPrep was used for every 10 mL of plasma. The plasma was then centrifuged at 2000 rpm for 15 min. The supernatant plasma layer was removed, and the monocytes isolated at the interface [42]. This method gave monocytes of >90% purity by CD14 staining. Normally 1 × 10^6^ monocytes were obtained from every 10 mL of blood. Isolated monocytes were then washed twice in 1% BSA/PBS and resuspended for the adhesion assays.

### 2.4. Isolation of Human LDL and Oxidation

Blood was taken from healthy volunteers. LDL (1.019 to 1.063 g/mL) was isolated from the plasma by sequential density ultracentrifugation at 4 °C, as described previously [43]. The LDL was oxidised either by storage in the dark for 3–6 months, or treated with 1 μM FeSO_4_ for 96 h at 4 °C. These methods give rise to minimally modified LDL, principally containing oxidised phospholipids. It is referred to as oxidised LDL (oxLDL).

### 2.5. Plate Sensitisation

A coating solution of 15 mM sodium carbonate with 0.05% sodium azide at pH 9.6 was employed for sensitising high binding 96 well plates (Merck, CLS9108). The coating proteins, human fibronectin (FN) (Merck), low endotoxin HSP60 (Enzo, Exeter, UK), and oxLDL, were added at 10 µg protein/mL, with 50 µL per well. The plates were then placed briefly on a vortex mixer to ensure even coating of the base of all wells, and incubated at 4 °C overnight, and washed four times in PBS. In assays with blood monocytes, a short blocking step in a 1% albumin-PBS solution was then additionally performed beforehand.

### 2.6. Fluorescent Static Solid Phase Adhesion Assay

Ninetysix-well plate fluorescent adhesion assays were performed with the sensitised plates to investigate the role of oxLDL and HSP60 in the induction of U937 cell and human monocyte adhesion, together with the identification of ligands involved by inhibition with antibodies and other agents. The antibodies are listed in Table 1, together with the non-immune immunoglobulin controls against which they are compared in the experiments. All the antibodies were murine monoclonals against human determinants, except a polyclonal rabbit anti human apolipoprotein B. In preparation for the assays, 5-(and 6)-carboxyfluorescein diacetate, succinimidyl ester (CFDA-SE; ThermoFisher C-1157, Waltham, MA, USA) was dissolved in DSMO to give a 10 mM stock solution. This was stored in aliquots at −20 °C or −80 °C, in the dark. To perform an assay, carboxyfluorescein stock (10 mM) was diluted to 100 µM in the cell suspension in medium with 5–10 × 10^6^ cells/ml. This was covered in foil, and incubated for 30′. The cells were washed three times in RPMI +10% FCS, and 50 μL added to the 96 well plates at a final concentration of 3.2 × 10^5^ cells/well. Also studied were the membrane active agents, nystatin, methyl β cyclodextrin (MCD) and dimethyl sulfoxide (DMSO) (Sigma-Aldrich, St. Louis, MO, USA). MCD and nystatin are cholesterol binding and depleting agents, and DMSO a lipid solvent. The wells had inhibitors added diluted in the same medium where required to a final total volume of 100 μL. Assays were done with at least triplicate replicates. The plates were incubated at 37 °C for one hour in a CO_2_ incubator. The wells were then washed three times. Aliquots of the original cell suspension were added to at least three unused wells. The plates were then counted in a Fluoroskan 96 well plate reading fluorescent spectrophotometer (ThermoFisher). The data are expressed as the percentage of input cells bound ± SEs, using a mean value of the wells filled with cell suspension as reference.

### 2.7. Surface Expression of Oxidised Phospholipid, MDA and HSP60 on HUVEC

Human umbilical vein endothelial cells (HUVEC), ref C12203, were obtained from Promocell, Heidelberg, Germany, or were gifts from colleagues. The cells were cultured as previously described [45], added to gelatin coated 96 well plates, and grown to confluency in medium with either 20% FCS or 20% normal human serum (NHS) for at least 18 h. Five replicates were used for each condition. They were then washed in PBS, fixed in 0.1% glutaraldehyde for 10 min, washed and blocked with 10% milk in PBS for 1 h. Primary antibodies to oxidised phospholipid (EO6), MDA and to HSP60 at appropriate dilutions, or the same concentrations of Ig controls were added to the plates and placed on a shaker for one hour. All subsequent stages were also on the shaker. The wells were then washed three times with 10% milk for 10 min, and then incubated with 1/2000 peroxidase conjugated rabbit anti mouse Ig in 10% milk for one hour at room temperature. They were then washed once more with 10% milk, and twice with PBS. A colour reaction for peroxidase was produced with o-phenylenediamine dihydrochloride (OPD) prepared from tablets (Sigma), and absorbance read at 450 nm.

### 2.8. Immunohistochemistry (IHC)

The avidin-biotin-complex (ABC) immunoperoxidase technique was used to observe endothelial expression of HSP60 and oxLDL in frozen atherosclerotic plaque specimens. Tissue sections were cut onto APES-coated slides and stored at −20 °C. Non-specific binding was inhibited with 5–10% serum of the same species as the chosen secondary antibody added for at least 10 min. Excess serum was tapped off, before addition of the primary antibody in PBS, and incubated for one hour, then washed twice with PBS. The details of all primary antibodies used in the study are given in Table 1.

The secondary antibody, complementary to the Ig species of the primary antibody and conjugated to biotin, was added, before slide incubation for 30 min and two-fold washing. Glucose oxidase or 0.3–1.0% hydrogen peroxide in PBS was added, incubated for 30 min and washed twice, to block corresponding intrinsic enzymes. Slides were then incubated for 30 min at 37 °C, before being washed twice. Preincubated avidin-biotin complex was added to the slides, before a 30 min incubation period and two-fold PBS washing. The slides were then flooded with diaminobenzidine 0.75 mg/mL and hydrogen peroxide 0.015% solution for 5–15 min, washed with tap water, counterstained with Mayer’s haematoxylin, and differentiated in acid alcohol for a few seconds. They were then dehydrated and mounted in DPX Mountant. Endothelial expression was quantitated as previously described [19].

### 2.9. Adhesion under Static and Flow Conditions

The effect of flow on adhesion of peripheral blood mononuclear cells (PBMC) to oxLDL was investigated using Ibidi μ-Slides VI slide chambers (Ibidi, Germany), as previously described [46]. PBMC were isolated from healthy volunteer donors by separation on a Histopaque (Sigma) gradient according to the manufacturer’s instructions. The Ibidi slides were coated overnight with oxLDL or native LDL (nLDL) at 10 μg/mL, or with a recombinant stable modified form of human galectin-9 (Gal 9) at 20 μg/mL as positive control [46]. This stable form of Gal-9 was obtained from GalPharma (Takamatsu, Japan). Gal-9 is highly susceptible to proteolytic degradation at the linker region. The stable form of Gal-9 is a mutant of native Gal-9 with a truncated linker region, which prevents proteolytic degradation whilst retaining all known activities of native Gal-9 [47,48]. PBMC were fed into the chambers at 10^6^ cells/mL at a shear rate of 1 dyne/cm^2^. For adhesion under flow, video sequences were taken after 7 min of flow: for static adhesion the flow rate was stopped for 15 min prior to recording fields for analysis. Images were then quantitated off-line using Image-Pro (Media Cybernetics, Rockville, MD, USA) software.

### 2.10. Data Analysis and Statistical Analysis

In the plate adhesion assay, Fluoroskan results were used to calculate percentage adhesion to the wells. These were averaged across the repeats and plotted as bar charts. All assays were performed with varied numbers of repeats, as indicated in figures. In some, mean values of all experiments are shown, in others typical results, as stated. Statistical analysis, by one-way analysis of variance (ANOVA), was performed using GraphPad Prism (Version 8), or by paired *t* tests. A *p*-value of <0.05 was deemed statistically significant. The standard error of repeats is shown on the graphs.

## 3. Results

### 3.1. Ox-LDL as a Monocytic Cell Adhesion Ligand, Actions of Inhibitory Antibodies

The adhesion assays used both monocytes isolated from donor blood, and cultured stimulated U937 cells, but as the U937 cells were more easily available, the bulk of the assays were performed with them. No major differences in behaviour between them were observed.

**Figure 1:**
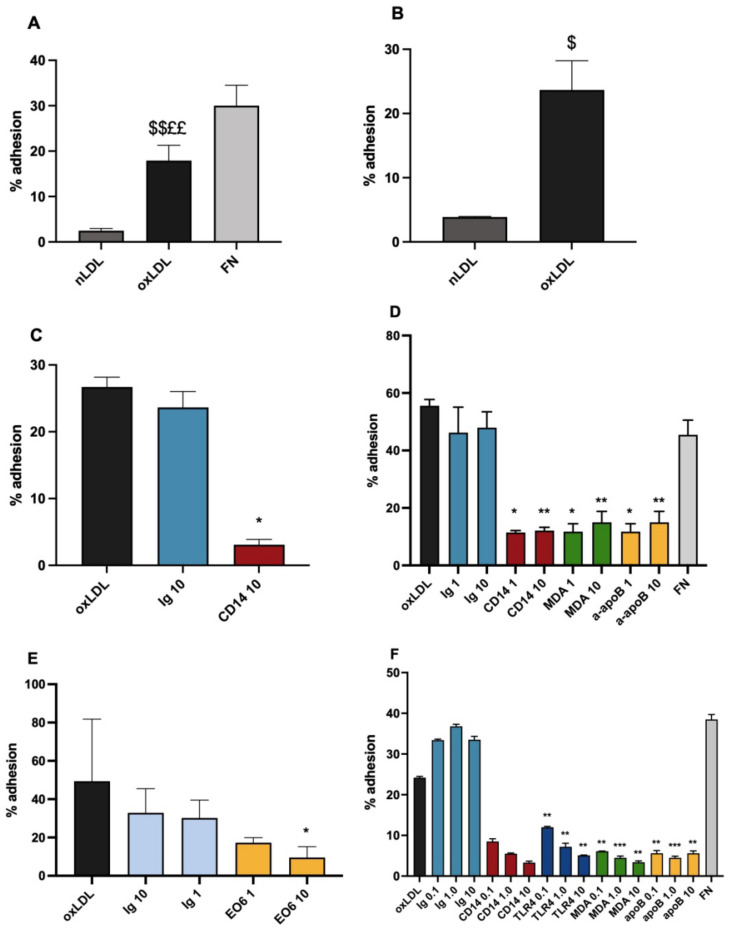
U937 cell and blood monocyte adhesion to oxidised LDL in a static adhesion assay. (**A**) Adhesion of U937 cells to native LDL (nLDL), oxidised LDL (oxLDL) and fibronectin (FN); means of six experiments (expts). (**B**) Monocyte adhesion to nLDL and oxLDL, one of two similar expts. (**C**) Inhibition of U937 adhesion to oxLDL by control immunoglobulin (Ig) or CD14 antibody, means of four similar expts. (**D**) Inhibition of adhesion of monocytes by antibodies to CD14, malondialdehyde (MDA), and apolipoprotein B (apoB). One of two similar MDA expts. (**E**) Inhibition of U937 adhesion to oxLDL by anti-oxidised phospholipid antibody (EO6), one of three similar expts. (**F**) Inhibition of U937 adhesion to oxLDL by antibodies to CD14, Toll like receptor 4 (TLR4), MDA and apoB, one of three similar TLR4 expts. Control Ig in C, D and F is UPC10, in E it is MOPC104E. Figures in inhibitor and control Ig legends are concentrations in μg/mL. Statistical analysis was by paired *t* tests and ANOVA; $ *p* < 0.05, $$ *p* < 0.01 compared to (v) nLDL; ££ *p* < 0.01, v FN; and * *p* < 0.05 ** *p* < 0.01, *** *p* < 0.001 v Ig control of same concentration.

Solid phase oxLDL was effective as a mononuclear cell adhesion ligand. Comparison was made with fibronectin (FN), a well established monocyte ligand, which binds to their α_5_β_1_ integrin receptor, and with nLDL. With U937 cells the adhesion to oxLDL was significantly higher than that observed with nLDL, but the adhesion to oxLDL was significantly lower than adhesion to fibronectin (FN) (Figure 1A). As was observed with U937, significantly higher numbers of blood monocytes adhered to oxLDL that nLDL (Figure 1B) but in contrast to U937, blood monocytes bound with a similar efficiency to both oxLDL and FN (Figure 1D). These results show that LDL oxidation induces the adhesion of both peripheral monocytes and U937 cells.

As CD14 has been implicated both as an oxLDL ligand, and in monocyte adhesion to endothelial cells [28], inhibition by a CD14 antibody was investigated. With U937 cells, UCHM1 gave dose related or near-complete inhibition of U937 adhesion to solid phase ox-LDL (Figure 1C), and there was a similar effect on monocytes (Figure 1D). With U937 cells, this inhibition is compared to the effect of the IgG2a isotype matched Ig control, UPC10, in four experiments, and was highly significant in all: the combined results are shown in Figure 1C. In Figure 1D it can be seen that the same strong inhibition by anti-CD14 compared to control Ig applies with monocyte adhesion. These results show that an antibody to CD14 is capable of interfering with the adhesion process, thus providing evidence that CD14 and the complexes with TLR4, in the raft membrane microdomains in which it is found, are involved in the adhesion interaction.

To further investigate the adhesion, the effects of antibodies against components of oxLDL and the putative CD14–TLR4 receptor complex were also studied. For oxidation products in oxLDL, the effects of antibodies to malondialdehyde and oxidised phospholipids were used. A monoclonal antibody to MDA, MDA2, gave effective inhibition of adhesion, both with monocytes and U937 cells, even to 0.1 μg/mL with the latter (Figure 1D,F). Phospholipids, which are present on the surface of the LDL particle oxidize readily, and so form ligands known to induce inflammatory reactions. They also show evidence of a role in the adhesion, as the monoclonal EO6 was inhibitory (Figure 1E). It is relevant to note that this antibody inhibited significantly more than the IgM control Ig MOPC104E, as IgM can give an appreciable level of non-specific binding to a plastic surface (manuscript in preparation). In addition, an antibody against native LDL, to apolipoprotein B was used. Interestingly, this also gave strong inhibition (Figure 1D,F), suggesting that the apolipoprotein had a significant role in the adhesion mechanism. However, this apolipoprotein can be in a modified state in oxLDL, and still maintain reactivity with antibodies.

To demonstrate that the CD14-TLR4 complex is involved, inhibition of adhesion by a TLR4 antibody was investigated. An antibody to TLR4 gave strong dose-related inhibition of U937 adhesion (Figure 1F). The involvement of this molecule is potentially important, as it is a major monocyte co-receptor with CD14, and implicated in transmembrane signalling.

### 3.2. HSP60 as a Monocytic Cell Adhesion Molecule, Actions of Inhibitory Antibodies

**Figure 2:**
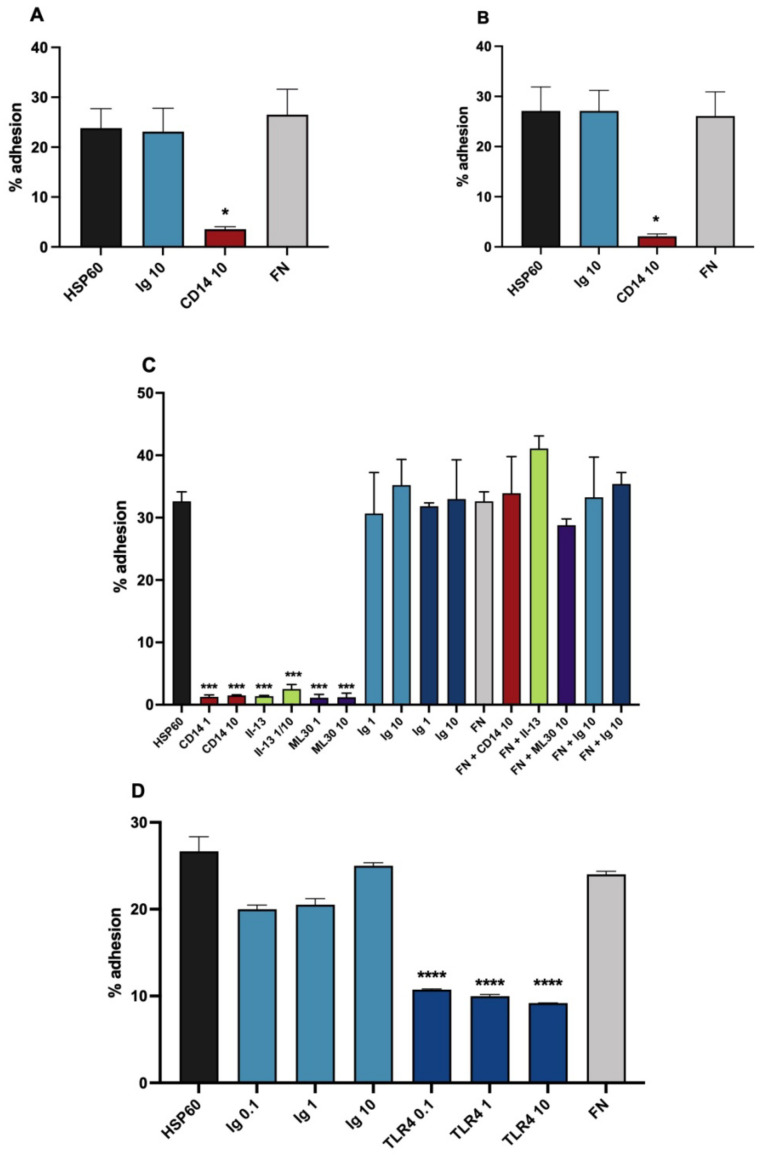
U937 cell and blood monocyte adhesion to heat shock protein 60 (HSP60) in a static adhesion assay. (**A**) U937 adhesion, inhibition by CD14, means of 3 expts. (**B**) Monocyte adhesion, inhibition by anti-CD14, means of four expts. UCHM1 antibody used in A an dB. (**C**) Monocyte adhesion, inhibition by anti-CD14 and HSP60 antibodies II-13 (neat supernatant and 1/10 dilution) and ML30; effects of the same antibodies on FN adhesion. One of two similar expts. (**D**) U937 adhesion, inhibition by TLR4 antibody HTA125, single expt. Control Ig is UPC10 in A, B and D, UPC10 and MOPC21 (dark blue) in C. Statistical analysis was by paired *t* tests and ANOVA, * *p* < 0.05, *** *p* < 0.001, **** *p* < 0.0001 v appropriate Ig control.

In a similar way to oxLDL, HSP60 coated wells gave effective adhesion of U937 cells and blood monocytes in the static assay. Further, the adhesion was likewise significantly inhibited by the CD14 antibody with both cell types (Figure 2A,B). To demonstrate the specificity of the adhesion to the HSP60 molecule, a panel of HSP60 antibodies was employed, all of which produced profound inhibition of the adhesion in the assay. Figure 2C shows the results with II-13, used as a hybridoma supernatant, and with ML30. A further antibody, LK1, gave similar results. Importantly, in Figure 2C, the same two antibodies were also investigated in fibronectin sensitized wells, in which they were completely without inhibitory activity, demonstrating specificity of their activity to HSP60 well sensitization. As further controls, the non-immune Igs UPC10 (IgG2a) and MOPC21 (IgG) were also without inhibitory activity. Again, as with oxLDL, it was important to show the role of the TLR4 in addition to CD14 in the receptor complex. Accordingly, inhibition by the TLR4 antibody was investigated, and highly significant inhibition of the adhesion was produced (Figure 2D), a result that was reproducible with blood monocytes.

### 3.3. Effects of Membrane Active Agents

**Figure 3:**
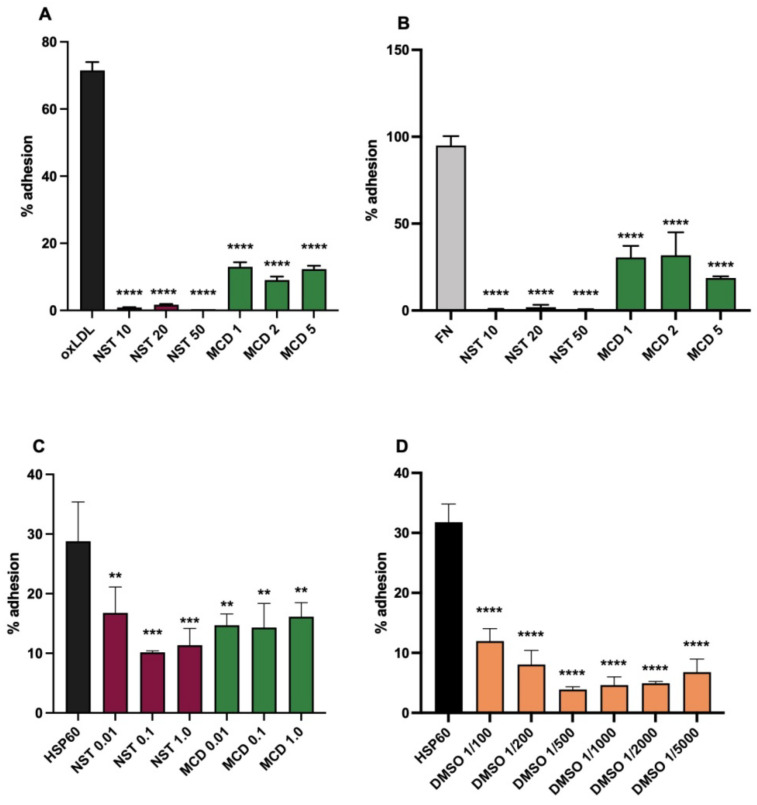
Inhibitory activity of membrane active agents on U937 adhesion. (**A**) The inhibition by raft disrupting agents nystatin (NST) and methyl β cyclodextrin (MCD) of adhesion to oxLDL at standard concentrations. (**B**) By NST and MCD to FN at standard concentrations. (**C**) By NST and MCD to HSP60 at low concentrations. (**D**) By dimethyl sulfoxide to HSP60, dilutions in assay stated. Concentrations of NST are given in μg/mL and MCD in mM. A total of three expts were done with each of oxLDL, FN and HSP60 as ligands. Statistical analysis by ANOVA, different from HSP60, ** *p* < 0.01, *** *p* < 0.001, **** *p* < 0.0001.

To investigate whether lipid raft domains were involved in the CD14/TLR4 dependent adhesion, the effects of the raft disrupting agents NST and MCD were studied on U937 cells, together with those of DMSO. At concentrations used previously for raft disruption, both NST and MCD strongly inhibited U937 adhesion to oxLDL (Figure 3A), and to fibronectin (Figure 3B). As these agents are cholesterol solvents, the effect on oxLDL adhesion could conceivably be explained by disruption of the oxLDL target, however the very similar effects on FN adhesion preclude this from being the major mechanism, and additionally indicates that the known integrin mediated adhesion of monocytic cell adhesion to FN is also likely to be dependent on membrane rafts. Further, CD14/TLR4 mediated adhesion was extremely sensitive to the action of nystatin and MCD, as these inhibitors were active in lower concentrations than usually used for raft disruption, seen with binding to HSP60 (Figure 3C). OxLDL gave similar results. Similarly, the changes in membrane structure caused by DMSO proved extremely active at inhibiting HSP60 adhesion, down to the lowest level tested of a 1/5000 dilution in the assay (2.8 mM DMSO) (Figure 3D). Together, these results suggest that the CD14/TLR4 dependent adhesion is highly sensitive to the fine structure of the monocytic cell membrane.

### 3.4. Variants of the Assay

**Figure 4:**
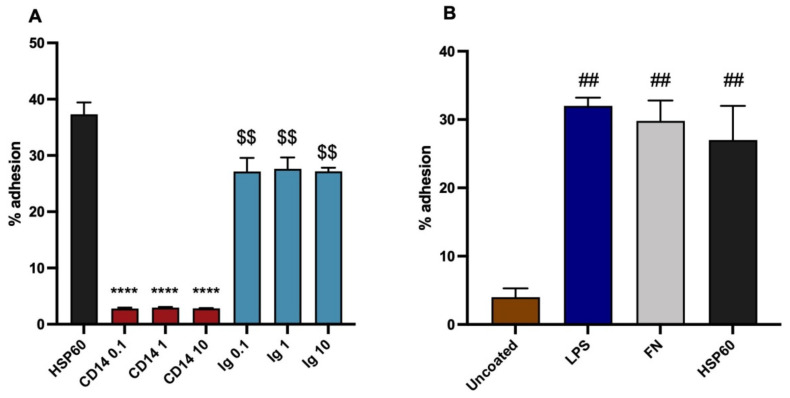
Variants on the adhesion assay. (**A**) Monocyte adhesion to HSP60 with cells suspended in 1% bovine serum albumin: inhibition by CD14 antibody or Ig control (UPC10). (**B**) Monocyte adhesion to lipopolysaccharide (LPS), comparison with FN or HSP60 as ligands. Statistical analysis by ANOVA; different from appropriate Ig control, **** *p* < 0.0001; different from HSP60, $$ *p* < 0.01; different from uncoated, ## *p* < 0.01.

It proved possible to perform the assay with the cells suspended in PBS with 1% bovine serum albumin, rather than in medium with 10% FCS. This demonstrated in Figure 4A, with monocyte adhesion to HSP60, with inhibition by CD14 antibody, compared to slight but significant inhibition by control Ig. The results are very similar to those with the standard method. This variation could be useful in avoiding any non-specific effects of serum proteins, which may exist.

In view of the ability of both HSP60 and LDL to bind LPS, it is relevant to determine whether LPS itself can act as a monocyte adhesion molecule. Therefore, an experiment was done in which it was coated similarly to the other ligands. It proved to be equally effective at binding monocytes as FN and HSP60 (Figure 4B). This result implies that there is a possibility that monocytic binding to both oxLDL and HSP60 might depend at least to some extent on the presence of bound LPS. LPS binding by LDL is a physiological interaction, however the low levels of adhesion of non-oxidised LDL found in this study demonstrate the changes on oxidation are by far the major contributors to the adhesion found with oxLDL. The situation with HSP60 is more complex, and is discussed below.

### 3.5. Comparison of PBMC Adhesion to oxLDL under Static and Flow Conditions

Blood PBMC were perfused through a flow chamber to allow determination of adhesion to oxLDL under both static and flow conditions, compared to galectin 9 [46] as the positive control. The monocyte isolation procedure was found not to exclude platelets, which adhered to the chambers under all conditions, including controls.

**Figure 5:**
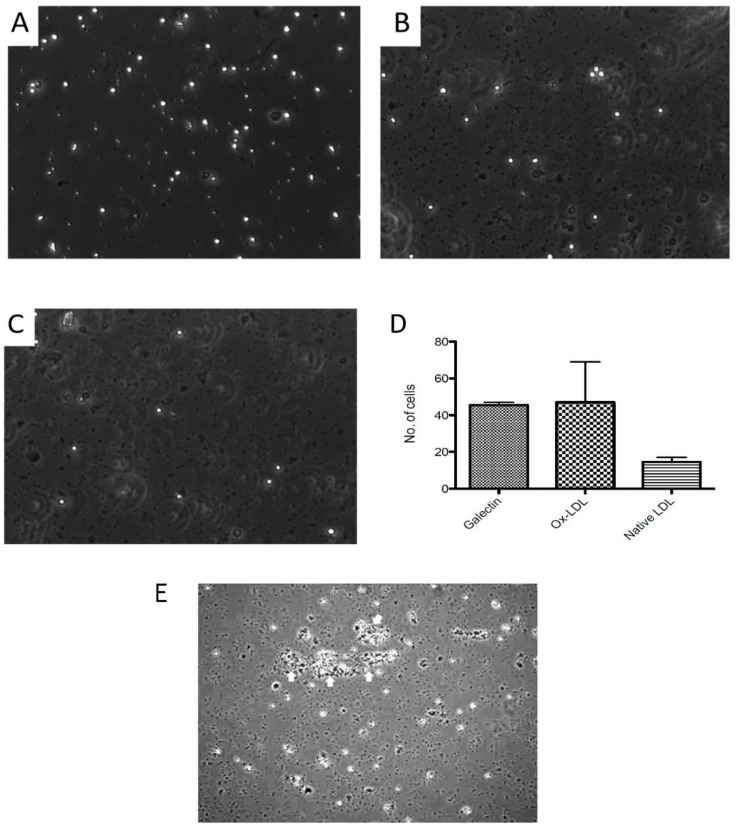
Static adhesion of peripheral blood mononuclear cells to oxidised LDL in a flow chamber. The cells were introduced into a flow chamber coated with ligands, measured under static conditions, viewed by phase contrast microscopy and quantitated. Coating: (**A**) human recombinant galectin 9, (**B**) oxidised LDL, (**C**) native LDL. (**D**) Quantitation of adhesion by image analysis of cells per area. (**E**) Formation of mononuclear cell- platelet aggregates (arrows) on adhesion to oxLDL. Four similar flow chamber expts were done, each under flow and static conditions.

Under static conditions, oxLDL allowed effective adhesion of the PBMC, similar to galectin-9 (Figure 5A,B), and was much less with native LDL (Figure 5C,D). However, under flow no adhesion to oxLDL was seen, despite galectin-9 recruiting and supporting adhesion. The presence of platelets resulted in an unexpected finding, in two of four experiments with different cell donors, aggregates of PBMC with platelets were present on static adhesion to oxLDL (Figure 5E), which were absent with the other ligands.

### 3.6. Expression of Oxidised Lipids and HSP60 on HUVEC

**Figure 6:**
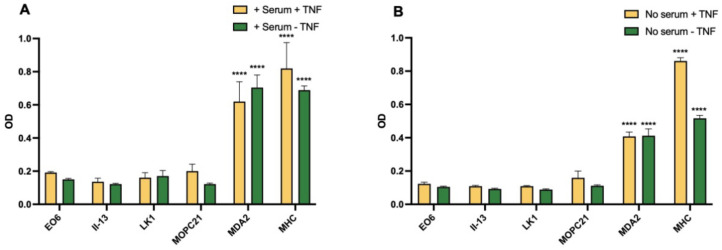
ELISA assay for the surface expression of oxidised lipids and HSP60 on HUVEC. (**A**) Cells cultured in 20% normal human serum with and without TNFα stimulation. (**B**) A second plate in same assay with culture in 20% FCS. Statistical analysis by ANOVA, different from appropriate MOPC21 (Ig) control, **** *p* < 0.0001. OD, optical density.

Experiments were done to detect oxidised lipids and HSP60 on HUVEC. The method did not include permeabilisation, so that surface expression relevant to adhesion was measured. Normal human serum (NHS) contains higher levels of lipoproteins than FCS, and overnight culture of HUVEC in 20% NHS was compared to 20% FCS, to provide a source of lipid to affect the endothelial cells. There was no significant increase in binding with the EO6 antibody to oxidised phospholipids compared to MOPC21 Ig controls in any of three experiments, whereas a control antibody to MHC-1 (Figure 6) or ICAM-1 gave a strong signal. By contrast, MDA was strongly positive. In one experiment which compared signals +/− NHS and +/− TNFα, giving four sets of data, MDA was strongly expressed under all these conditions, with similar results (Figure 6). The variations in conditions did not add any further relevant information, but all served to demonstrate the surface expression of MDA. In a further three similar experiments, no significant binding of the HSP60 antibody panel compared to Ig control was found.

### 3.7. Oxidised Lipids and HSP60 in the Endothelium of Human Atherosclerotic Plaques

**Figure 7:**
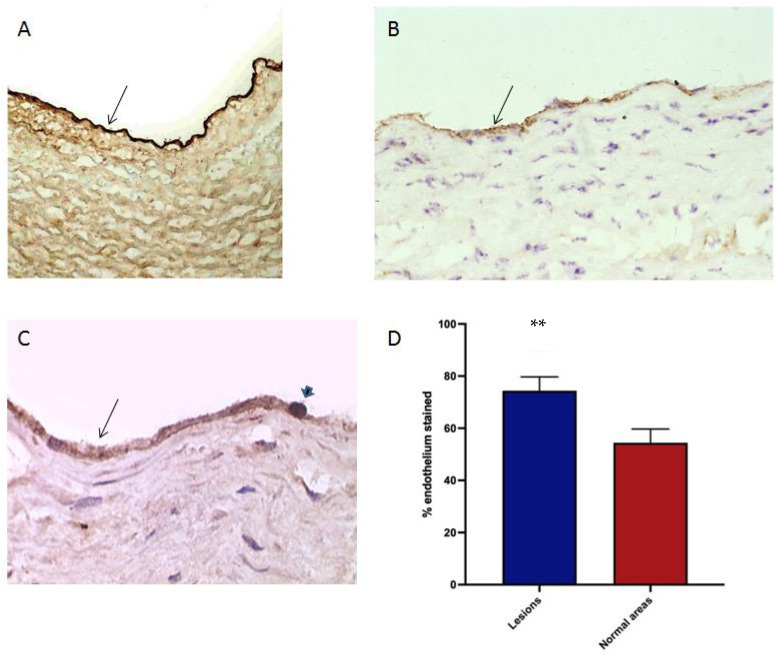
LDL, HSP60 and MDA in human arterial normal and atherosclerotic endothelium. (**A**) Native LDL in normal arterial wall, apoB antibody. (**B**) HSP60 in endothelium of atherosclerotic plaque, ML30 antibody. (**C**) MDA in atherosclerotic endothelium, MDA2 antibody. (**D**) Image analysis of MDA2 reactivity in atherosclerotic (lesions) and normal endothelium, analysis of 10 coronary or carotid arteries. Arrows—endothelium, arrowhead—probable mononuclear cell in traffic: ** *p* < 0.01, paired *t* test.

To determine the possible target molecules for the monocyte- oxidised receptor adhesion in the endothelium of atherosclerotic human arteries, IHC was done on specimens of coronary and carotid vessels for LDL and oxidation epitopes. It is interesting that the arterial endothelium of large arteries is heavily laden with native LDL: this was found universally in a panel of 20 vessels, with no apparent difference between normal and atherosclerotic regions (Figure 7A). This correlates with the existence of a specific transport mechanism [3,4].

Oxidised phospholipid was sought in the endothelium by EO6 IHC. None was found in 15 atherosclerotic arteries, despite high levels in plaque macrophages. By contrast, endothelial reactivity for MDA was extensive in atherosclerotic vessels (Figure 7B), with an increase over relatively normal areas in the same vessels, which were without significant thickening of the intima or macrophage infiltration. Comparison was made by image analysis, demonstrating a significant increase in the atherosclerotic areas (Figure 7D).

## 4. Discussion

This study focuses on the demonstration that oxLDL, and HSP60 can act as adhesion ligands for monocytes under static conditions; the adhesion being mediated at least in part by interactions through their CD14/TLR4 receptor complex. It has shown that PMA stimulated U937 cells are an excellent model for blood monocytes in adhesion studies with almost identical results, so it is appropriate for the results to be discussed as those for monocytes. During the course of the work, evidence has accumulated that MDA-modified adducts may be a monocyte adhesion target. MDA can also be an adduct onto molecules other than those in LDL, which could widen its relevance. Western blotting of HUVEC would be useful to identify the modified proteins. Only one similar previous report is known which showed that under static conditions, that macrophages adhere to oxLDL via scavenger receptors [49]: it is discussed below. Although the ability of oxLDL to activate monocytes is well known, the adhesive consequences of the interaction have been little considered. They could be important in the recruitment of monocytes from the blood to the developing atherosclerotic plaque if the relevant oxidised lipid targets are presented on the interior surface of the arterial wall, either on endothelial cells, other cells, or on non-cellular matrix.

Adhesion under static conditions can contribute to the overall process of monocyte– endothelial adhesion, for example it is the mechanism of the important endothelial adhesion molecule ICAM-1 [50]. In atherosclerosis, static adhesion interactions could synergise with P-selectin, which is active under flow, and is highly expressed in atherosclerotic plaques [51]. The previous static adhesion study [49] showed that macrophages adhered to solid phase oxLDL through both scavenger receptor A, and the scavenger receptor CD36. The CD36 activated the macrophages to produce hydrogen peroxide. Similarly, the scavenger receptor CD36 is present on monocytes, and is known as a platelet adhesion receptor for collagen [52] and thrombospondin-1 [53]. The role of scavenger receptors in leukocyte adhesion has been reviewed quite recently [54].

The results here have shown that under static conditions, oxLDL is an effective monocyte adhesion molecule, similar to FN in its ability to bind to the cells. This ability lies specifically in the oxidation, as native LDL bound much less effectively. The mechanisms of adhesion of monocytes to oxLDL and HSP60 were investigated by inhibition with specific antibodies, and controlled by the use of non-immune Igs, a recognised approach used in many in vitro studies, which is also effective in vivo. Adhesion was effectively inhibited by the antibodies to all the candidate molecules investigated, but much less by the control Igs. With the oxLDL target, the CD14 antibody demonstrated the role of this receptor. As CD14 is constrained to rafts by its GPI anchor, it provides evidence for the involvement of lipid rafts in the adhesion mechanism. The activity of the TLR4 antibody also confirmed this molecule’s involvement in the adhesion receptor complex. Inhibition by the MDA antibody correlated with its known role in monocyte adhesion to oxLDL via scavenger receptors, as found previously [49]. Inhibition by an apoB antibody of adhesion to the oxLDL target, although apoB is a normal part of the LDL structure, can be explained by its modification by malondialdehyde forming an adduct, and so could become a ligand. It is however an open question whether the inhibitory antibody has to be against an adhesion ligand, or could just be in the close proximity and cause inhibition by steric hindrance.

Ligand binding to CD14 receptor complexes recruits and modulates receptors in the complex, which include several Fc receptors [55]. Further, such complexes can undergo endocytosis following CD14 ligation [56]. They can also after Fc receptor engagement, which might occur through cross-linking following the addition of the experimental antibodies to adhesion ligands in the complex, when the Fc of the added antibody could bind to Fc receptors in the complex. Hence, antibodies to CD14 and other receptors in the complex might cause inactivation through its loss from the cell membrane, with CD14 antibodies even possibly by two mechanisms. Such Fc interactions might also explain the unexpected commonly seen mild inhibitory effects of control Igs in the adhesion experiments (e.g., in Figure 4A). Binding of experimental antibodies or controls with Fc receptors could be enhanced if they contained Ig aggregates, which can bind avidly to Fc receptors: a few experiments failed when control Ig inhibition became very large. Overall, it seems possible that the monocyte adhesion receptor- raft complex, containing multiple individual adhesion receptors, may behave in an organized integrated fashion. In this way it could resemble the immunological synapse involved in T lymphocyte- antigen presenting cell interactions [57].

Further evidence that monocyte adhesion involved lipid rafts is shown here by the great sensitivity of all the adhesion interactions investigated using the raft disrupting agents MCD and nystatin. The adhesion targets so affected included FN. This shows that with an oxLDL target these agents were not acting primarily by disrupting the oxLDL through their action as cholesterol solvents, because the results were very similar with both targets. Previously, MCD had also been shown to cause endothelial cells to reduce adhesion molecule expression and the adhesion of monocytes [58], and to inhibit monocyte adhesion to endothelial cells stimulated with LPS or oxLDL [59], although the involvement of lipid rafts in these events was not investigated. In addition, the sensitive disruption shown here of adhesion to FN, which binds primarily to α_5_β_1_ integrin, confirms that raft structure is required for integrin mediated adhesion, as determined previously [60].

A further factor which may aid in the adhesion of monocytes to oxLDL in the assay is the ability of oxLDL to activate the cells. The interaction induces activation of integrin adhesion to endothelial cells [11,61], which might also lead to increased adherence to the plastic of the plate wells, although the mechanisms of this interaction are poorly defined. OxLDL mediated monocyte activation via CD14 and TLR4 has also been shown to induce cytokine secretion [16]. The abilities of statins to inhibit oxLDL effects on monocytes are well documented [62,63,64,65]. It would therefore be of considerable interest to test their effects in the oxLDL adhesion assay: inhibition could add another arm to their pleiotropic beneficial effects.

The sensitive inhibition of monocyte adhesion seen with DMSO was studied following previous work on a different cell–cell interaction, lymphocyte mediated antibody dependent cellular cytotoxicity (R.N.P., unpublished results). This was also very effectively inhibited. Therefore, the profound effects that DMSO has on leukocyte cell membranes [25,26,66] are able to modify the cells’ abilities at cell–cell contact. Consequently the dissolving of potential inhibitory agents in DMSO for study in such cellular interactions has to be avoided. Importantly, DMSO has potential for the therapy of atherosclerosis, as it was shown to inhibit the disease in a rabbit model without lowering blood cholesterol [67]. Further, extensive studies have shown low toxicity in man, particularly at lower doses [68]. A clinical trial could be justified.

This study has shown that in a similar manner to oxLDL, solid phase HSP60 caused potent monocyte adhesion. However, the significance of this interaction is qualified by the likely presence of LPS in the commercial samples of HSP60 that were used, even though described as having low endotoxin when purchased some years ago [38]. With ox-LDL, the evidence that native LDL from the same source binds monocytes much less effectively shows that the adhesion is likely to depend on oxidation products, and not LPS. However, a similar argument does not apply to HSP60, and LPS itself was effective at binding monocytes. As HSP60 does not circulate to the extent of LDL, it is less likely to acquire LPS in vivo. Hence, the in vivo relevance of this adhesion is in doubt, despite the demonstrated frequent presence of HSP60 in the endothelial cells of atherosclerotic lesions, and the knowledge that it can be present at their cell surfaces [34]. In the HSP60 adhesion assay, comparison of the inhibitory activity of antibodies against the other heat shock proteins, such as HSP90, that are not surface- expressed could be a valuable negative control.

The principal importance of monocyte adhesion to oxLDL particles in vivo may be adhesion of monocytes to exposed sub-endothelial oxLDL following endothelial denudation in atherosclerosis. Tissue samples have shown that oxidised phospholipids are plentiful in the human atherosclerotic intima, including in subendothelial fatty streak lesions [69], furthermore both endothelial cells and macrophages can induce the local oxidation of LDL [70]. LDL is plentiful in the arterial intima, and the accumulation of oxLDL is a consequence of the active traffic of LDL into the arterial wall, seen here by IHC, combined with labile nature of LDL particles. It is worth noting that the endothelial LDL transporter, SR-B1, is down-regulated by oestrogens [71], which might be involved in the protection of pre-menopausal women from atherosclerotic disease. Once formed in the arterial intima, it is possible that oxLDL bound to matrix, or derived phospholipids, could mediate monocyte adhesion to exposed non-endothelial intima, and explain the CD14 dependent adhesion seen previously with tissue sections of atherosclerotic arteries [29]. Loss of endothelial integrity, potentially exposing intimal oxLDL to the blood, is an important antecedent to thrombosis [72]. It was interesting and unexpected that synergy between monocyte and platelet adhesion was observed in the flow chamber experiments, through the accidental presence of platelets in the monocyte preparation, and gave rise to aggregates of monocytes and platelets. The interactions between monocytes and platelets are highly relevant to arterial thrombosis, as monocytes are present in thrombi [73]. In this context, these interactions are likely to result from the simultaneous activation of the monocytes and the platelets on oxLDL, with the platelet CD36 receptor likely to be involved in their activation [74]. The aggregation is mediated by interactions between the platelets and the monocytes, particularly by enhanced platelet P-selectin interaction with the monocyte PSGL-1 ligand [75,76]. Platelet– monocyte interaction enhances monocyte production of tissue factor and fibrin generation [77,78]. In these ways, monocyte adhesion to exposed intimal LDL oxidation products could exacerbate the development of thrombosis. Furthermore, circulating monocyte-platelet aggregates have been associated with acute coronary syndromes [79]. This potential mechanism could enhance the value of targeting monocytes and oxLDL in the prevention of thrombosis, in addition to atherosclerosis.

A further mechanism may allow oxLDL to bind to the glycocalyx of endothelial cells. Human arterial endothelial cells secrete an endothelial cell specific lipoprotein lipase that is found in the surrounding cell glycocalyx [80]. It has been shown to bind oxLDL with high affinity, and could allow the concentration of oxLDL around endothelial cells [81]. Further, lipoprotein lipase has been found to be a monocyte adhesion ligand, but without known involvement of oxLDL [82]: possibly bound oxLDL can act as a target for monocyte adhesion.

There is an analogous potential involvement of oxLDL in adherence of monocytes to the LOX-1 scavenger receptor on endothelial cells. LOX-1 has been implicated as a monocyte adhesion molecule [83], again oxLDL could be binding to it, particularly as experimentally oxLDL in the medium was inhibitory. It is tempting to think of oxLDL as the filling in an adhesion sandwich in both instances.

It is also likely that macrophages in plaques will adhere to oxLDL, either via CD14/TLR4 or scavenger receptors. Within the body of the atherosclerotic intima, oxLDL is present bound to matrix. This could enhance through the adhesion, the retention and localization of macrophages that have entered by traffic from the blood. Ox-LDL would also activate them. These mechanisms could exacerbate the disease, and aid the focal development of an atherosclerotic plaque, particularly by concentrating the inflammatory actions of the macrophages [2].

The inhibition of adhesion to oxLDL found with the MDA2 antibody supports previous findings that MDA adducts are targets for monocyte adhesion via the CD36 receptor. CD36 was not investigated, as initially we were not anticipating this finding. This adhesion raises the possibility that monocytes might bind to the high levels of MDA adducts present in atherosclerotic endothelium. MDA adducts were detectable by ELISA on HUVEC, suggesting that they can be present at the endothelial cell membrane, as the cells in the assay were fixed but not permeabilised. Surface expression is clearly necessary for adhesion. As this evidence is indirect, further work is required to support this hypothesis. In addition, in animal models, an atherogenic diet or immunization induced MDA specific antibodies [84], which might enhance arterial inflammation and monocyte adhesion. CD36 is also a worthwhile target for further investigation in the treatment of cardiovascular disease, as CD36 low molecular weight inhibitors reduced both atherosclerosis and metabolic abnormalities in a leptin and LDL receptor double knockout mouse model [85].

The situation with CD14/TLR4 dependent adhesion to endothelial cells is less clear. Previous work has suggested that CD14 may be implicated in the adhesion of monocytes to endothelial cells [28] and to the atherosclerotic arterial wall [29]. Consequently we attempted some further experiments on CD14 dependent monocyte adhesion to HUVEC, but they were not technically successful. However, our results with cultured TNFα stimulated HUVEC gave no significant signals in ELISA assays using EO6 antibody reactive with oxidised phospholipid. Human atherosclerotic arteries were also investigated for the presence of oxidised epitopes by IHC. In our work, positive signals with EO6 were absent from the endothelial cells of lesions by the avidin-biotin complex technique, however a previous study by Gargalovic et al. [86] included an image (Figure 3D in that paper) that showed limited but distinct positivity at the luminal surface of the shoulder region of a human plaque, although it was not mentioned. This staining was accompanied by the inflammatory transcription factor ATF3 that can be induced by oxidised phospholipid. The positive cell type was not identified, but might have been endothelial cells or trafficking monocytes, either of which could be a site for further monocyte adhesion. Overall, our results have not provided good evidence to support oxidised phospholipids being targets for CD14 dependent adhesion to endothelial cells, but the IHC method might have lacked sensitivity, and TNFα might not have been the appropriate stimulant to mimic conditions in the arterial wall. However, it seems likely there is little oxidised phospholipid in the human atherosclerotic endothelium.

This study highlights the importance of some new avenues for therapy of atherosclerotic cardiovascular disease. The CD14-TLR2/4 receptor interaction with oxidised phospholipids has shown promise as a target, demonstrated by the development of a family of active oxidised phospholipid analogues, named lecinoxoids [87]. In a hyperlipidaemic rabbit atherosclerosis model, the lead compound VB-201 caused 50% reduction in lesions [87]. Similar results were obtained in the apoE knockout mouse atherosclerosis model [88]. These actions were despite no effect on the hyperlipidaemia. The lecinoxoids have been shown to inhibit CD14 and TLR2/4 dependent monocyte activation and inflammatory signalling [87]. It would be interesting to determine their effects on monocyte adhesion to oxLDL.

Importantly, cyclodextrins have been found recently to have anti-atherosclerotic activity in animal models [89,90,91,92]. Remarkably, in a mouse model, lesions were found to regress even with a continued atherogenic diet [90]. Multiple mechanisms of atherosclerosis amelioration have been identified:- cellular cholesterol efflux [93], decreased complement activation by cholesterol crystals [94,95], and an anti-inflammatory action mediated by simulation of the LXR transcription factor [90]. The effects of raft disruption have received less attention in this therapeutic action, although they could underlie some of these other mechanisms. Indeed, the cyclodextrins could have a major therapeutic advantage by acting in all these ways, bringing to mind the pleiotropic effects of the statins [96].

Overall, this study has provided a direct connection between the oxidation of lipid, known to be central to atherosclerosis, and monocyte adhesion, also known to be essential for this fundamentally inflammatory disease. The functional relevance of the mechanisms uncovered is already well supported by animal models, together offering exciting new possibilities for prophylaxis and therapy.

## Figures and Tables

**Table 1:** Antibodies.

Primary Antibodies	Immunoglobulin Class	Human Target Antigen	Supplier
EO6	IgM	Oxidised phospholipid	Avanti
HTA125	IgG_2a_	TLR4	BioRad
II-13	IgG_2a_	HSP60	Abcam
LK-1	IgG_1_	HSP60	Merck/Sigma
MDA2	IgG_2a_	Malondialdehyde	Abcam
ML30	IgG_1_	HSP60	Reference [44]
MOPC104E	IgM	Negative control	Merck/Sigma
MOPC21	IgG_1_	Negative control	Merck/Sigma
polyclonal	-	Apolipoprotein B	Dako/Invitrogen
UCHM1	IgG_2a_	CD14	BioRad
UPC10	IgG_2a_	Negative control	Merck/Sigma
W6/32	IgG_2a_	MHC/HLA Class 1	Abcam

## Data Availability

The data that support the findings of this study are available from the corresponding author, R.N.P., upon reasonable request.

## References

[1] Libby P., Ridker P.M., Hansson G.K. (2009). Inflammation in Atherosclerosis: From Pathophysiology to Practice. J. Am. Coll. Cardiol..

[2] Poston R.N. (2019). Atherosclerosis: Integration of its pathogenesis as a self-perpetuating propagating inflammation: A review. Cardiovasc. Endocrinol. Metab..

[3] Armstrong S.M., Sugiyama M.G., Fung K.Y., Gao Y., Wang C., Levy A.S., Azizi P., Roufaiel M., Zhu S.-N., Neculai D. (2015). A novel assay uncovers an unexpected role for SR-BI in LDL transcytosis. Cardiovasc. Res..

[4] Zhang X., Sessa W.C., Fernández-Hernando C. (2018). Endothelial Transcytosis of Lipoproteins in Atherosclerosis. Front. Cardiovasc. Med..

[5] Watson A.D., Leitinger N., Navab M., Faull K.F., Hörkkö S., Witztum J.L., Palinski W., Schwenke D., Salomon R.G., Sha W. (1997). Structural identification by mass spectrometry of oxidized phospholipids in minimally oxidized low density lipoprotein that induce monocyte/endothelial interactions and evidence for their presence in vivo. J. Biol. Chem..

[6] Friedman P., Hörkkö S., Steinberg D., Witztum J.L., Dennis E.A. (2002). Correlation of Antiphospholipid Antibody Recognition with the Structure of Synthetic Oxidized Phospholipids. J. Biol. Chem..

[7] Palinski W., Ylä-Herttuala S., Rosenfeld M.E., Butler S.W., ASocher S., Parthasarathy S., Curtiss L.K., Witztum J.L. (1990). Antisera and monoclonal antibodies specific for epitopes generated during oxidative modification of low density lipoprotein. Arterioscler. Off. J. Am. Heart Assoc. Inc..

[8] Skålén K., Gustafsson M., Rydberg E.K., Hultén L.M., Wiklund O., Innerarity T.L., Borén J. (2002). Subendothelial retention of atherogenic lipoproteins in early atherosclerosis. Nature.

[9] Simionescu N., Vasile E., Lupu F., Popescu G., Simionescu M. (1986). Prelesional events in atherogenesis. Accumulation of extracellular cholesterol-rich liposomes in the arterial intima and cardiac valves of the hyperlipidemic rabbit. Am. J. Pathol..

[10] Fukuchi M., Watanabe J., Kumagai K., Baba S., Shinozaki T., Miura M., Kagaya Y., Shirato K. (2002). Normal and Oxidized Low Density Lipoproteins Accumulate Deep in Physiologically Thickened Intima of Human Coronary Arteries. Lab. Investig..

[11] Cole A.L., Subbanagounder G., Mukhopadhyay S., Berliner J.A., Vora D.K. (2003). Oxidized phospholipid-induced endothelial cell/monocyte interaction is mediated by a cAMP-dependent R-Ras/PI3-kinase pathway. Arterioscler. Thromb. Vasc. Biol..

[12] Murohara T., Scalia R., Lefer A.M. (1996). Lysophosphatidylcholine promotes P-selectin expression in platelets and endothelial cells. Possible involvement of protein kinase C activation and its inhibition by nitric oxide donors. Circ. Res..

[13] Vora D.K., Fang Z.T., Liva S.M., Tyner T.R., Parhami F., Watson A.D., Drake T.A., Territo M.C., Berliner J.A. (1997). Induction of P-selectin by oxidized lipoproteins. Separate effects on synthesis and surface expression. Circ. Res..

[14] Morgan J., Smith J.A., Wilkins G.M., Leake D.S. (1993). Oxidation of low density lipoprotein by bovine and porcine aortic endothelial cells and porcine endocardial cells in culture. Atherosclerosis.

[15] Lamb D.J., Mitchinson M.J., Leake D.S. (1995). Transition metal ions within human atherosclerotic lesions can catalyse the oxidation of low density lipoprotein by macrophages. FEBS Lett..

[16] Chávez-Sánchez L., Chávez-Rueda K., Legorreta-Haquet M.V., Zenteno E., Ledesma-Soto Y., Montoya-Díaz E., Tesoro-Cruz E., Madrid-Miller A., Blanco-Favela F. (2010). The activation of CD14, TLR4, and TLR2 by mmLDL induces IL-1β, IL-6, and IL-10 secretion in human monocytes and macrophages. Lipids Health Dis..

[17] Duewell P., Kono H., Rayner K.J., Sirois C.M., Vladimer G., Bauernfeind F.G., Abela G.S., Franchi L., Nuñez G., Schnurr M. (2010). NLRP3 inflammasomes are required for atherogenesis and activated by cholesterol crystals. Nature.

[18] Miller Y.I., Shyy J.Y. (2017). Context-Dependent Role of Oxidized Lipids and Lipoproteins in Inflammation. Trends Endocrinol. Metab..

[19] Poston R.N., Haskard D.O., Coucher J.R., Gall N.P., Johnson-Tidey R.R. (1992). Expression of intercellular adhesion molecule-1 in atherosclerotic plaques. Am. J. Pathol..

[20] Reape T.J., Groot P.H. (1999). Chemokines and atherosclerosis. Atherosclerosis.

[21] Yin M., Li C., Jiang J., Le J., Luo B., Yang F., Fang Y., Yang M., Deng Z., Ni W. (2021). Cell adhesion molecule-mediated therapeutic strategies in atherosclerosis: From a biological basis and molecular mechanism to drug delivery nanosystems. Biochem. Pharmacol..

[22] Ciesielska A., Matyjek M., Kwiatkowska K. (2020). TLR4 and CD14 trafficking and its influence on LPS-induced pro-inflammatory signaling. Cell. Mol. Life Sci..

[23] Triantafilou M., Triantafilou K. (2002). Lipopolysaccharide recognition: CD14, TLRs and the LPS-activation cluster. Trends Immunol..

[24] Giocondi M.-C., Milhiet P.E., Dosset P., Le Grimellec C. (2004). Use of Cyclodextrin for AFM Monitoring of Model Raft Formation. Biophys. J..

[25] Gironi B., Oliva R., Petraccone L., Paolantoni M., Morresi A., Del Vecchio P., Sassi P. (2019). Solvation properties of raft-like model membranes. Biochim. Biophys. Acta BBA-Biomembr..

[26] De Ménorval M.-A., Mir L.M., Fernández M.L., Reigada R. (2012). Effects of Dimethyl Sulfoxide in Cholesterol-Containing Lipid Membranes: A Comparative Study of Experiments In Silico and with Cells. PLoS ONE.

[27] Płóciennikowska A., Hromada-Judycka A., Borzęcka K., Kwiatkowska K. (2015). Co-operation of TLR4 and raft proteins in LPS-induced pro-inflammatory signaling. Cell. Mol. Life Sci..

[28] Beekhuizen H., Blokland I., Tilburg A.J.C.-V., Koning F., Van Furth R. (1991). CD14 contributes to the adherence of human monocytes to cytokine-stimulated endothelial cells. J. Immunol..

[29] Poston R.N., Johnson-Tidey R.R. (1996). Localized adhesion of monocytes to human atherosclerotic plaques demonstrated in vitro: Implications for atherogenesis. Am. J. Pathol..

[30] Stocker R., Keaney J.F. (2004). Role of Oxidative Modifications in Atherosclerosis. Physiol. Rev..

[31] Marchio P., Guerra-Ojeda S., Vila J.M., Aldasoro M., Victor V.M., Mauricio M.D. (2019). Targeting Early Atherosclerosis: A Focus on Oxidative Stress and Inflammation. Oxid. Med. Cell Longev..

[32] Kol A., Lichtman A.H., Finberg R.W., Libby P., Kurt-Jones E.A. (2000). Cutting Edge: Heat Shock Protein (HSP) 60 Activates the Innate Immune Response: CD14 Is an Essential Receptor for HSP60 Activation of Mononuclear Cells. J. Immunol..

[33] Ohashi K., Burkart V., Flohé S., Kolb H. (2000). Cutting Edge: Heat Shock Protein 60 Is a Putative Endogenous Ligand of the Toll-Like Receptor-4 Complex. J. Immunol..

[34] Xu Q., Schett G., Seitz C.S., Hu Y., Gupta R.S., Wick G. (1994). Surface staining and cytotoxic activity of heat-shock protein 60 antibody in stressed aortic endothelial cells. Circ. Res..

[35] Soltys B.J., Gupta R.S. (1997). Cell surface localization of the 60 kDa heat shock chaperonin protein (hsp60) in mammalian cells. Cell Biol. Int..

[36] Xu Q., Kleindienst R., Waitz W., Dietrich H., Wick G. (1993). Increased expression of heat shock protein 65 coincides with a population of infiltrating T lymphocytes in atherosclerotic lesions of rabbits specifically responding to heat shock protein 65. J. Clin. Investig..

[37] Kleindienst R., Xu Q., Willeit J., Waldenberger F.R., Weimann S., Wick G. (1993). Immunology of atherosclerosis. Demonstration of heat shock protein 60 expression and T lymphocytes bearing alpha/beta or gamma/delta receptor in human atherosclerotic lesions. Am. J. Pathol..

[38] Gao B., Tsan M.F. (2003). Recombinant Human Heat Shock Protein 60 Does Not Induce the Release of Tumor Necrosis Factor α from Murine Macrophages. J. Biol. Chem..

[39] Levels J.H.M., Abraham P.R., Ende A.V.D., van Deventer S.J.H. (2001). Distribution and Kinetics of Lipoprotein-Bound Endotoxin. Infect. Immun..

[40] Shao B., Munford R.S., Kitchens R.L., Varley A.W. (2012). Hepatic uptake and deacylation of the LPS in bloodborne LPS-lipoprotein complexes. Innate Immun..

[41] Pedrinaci S., Ruiz-Cabello F., Gomez O., Collado A., Garrido F. (1990). Protein kinase C-mediated regulation of the expression of CD14 and CD11/CD18 in U937 cells. Int. J. Cancer.

[42] Tsouknos A., Nash G.B., Rainger G.E. (2003). Monocytes initiate a cycle of leukocyte recruitment when cocultured with endothelial cells. Atherosclerosis.

[43] Wilkins G.M., Leake D.S. (1994). The effect of inhibitors of free radical generating-enzymes on low-density lipoprotein oxidation by macrophages. Biochim. Biophys. Acta.

[44] Evans D.J., Norton P., Ivanyi J. (1990). Distribution in tissue sections of the human groEL stress-protein homologue. APMIS.

[45] Siow R.C.M. (2012). Culture of Human Endothelial Cells from Umbilical Veins. Methods Mol. Biol.

[46] Iqbal A.J., Krautter F., Blacksell I.A., Wright R.D., Austin-Williams S.N., Voisin M., Hussain M.T., Law H.L., Niki T., Hirashima M. (2022). Galectin-9 mediates neutrophil capture and adhesion in a CD44 and β2 integrin-dependent manner. FASEB J..

[47] Nishi N., Itoh A., Fujiyama A., Yoshida N., Araya S.-I., Hirashima M., Shoji H., Nakamura T. (2005). Development of highly stable galectins: Truncation of the linker peptide confers protease-resistance on tandem-repeat type galectins. FEBS Lett..

[48] Nishi N., Abe A., Iwaki J., Yoshida H., Itoh A., Shoji H., Kamitori S., Hirabayashi J., Nakamura T. (2008). Functional and structural bases of a cysteine-less mutant as a long-lasting substitute for galectin-1. Glycobiology.

[49] Maxeiner H., Husemann J., Thomas C.A., Loike J.D., El Khoury J., Silverstein S.C. (1998). Complementary roles for scavenger receptor A and CD36 of human monocyte-derived macrophages in adhesion to surfaces coated with oxidized low-density lipoproteins and in secretion of H_2_O_2_. J. Exp. Med..

[50] Nourshargh S., Alon R. (2014). Leukocyte Migration into Inflamed Tissues. Immunity.

[51] Johnson-Tidey R.R., McGregor J.L., Taylor P.R., Poston R.N. (1994). Increase in the adhesion molecule P-selectin in endothelium overlying atherosclerotic plaques. Coexpression with intercellular adhesion molecule-1. Am. J. Pathol..

[52] Tandon N.N., Kralisz U., AJamieson G. (1989). Identification of glycoprotein IV (CD36) as a primary receptor for platelet-collagen adhesion. J. Biol. Chem..

[53] Silverstein R.L., Febbraio M. (2000). CD36 and atherosclerosis. Curr. Opin. Lipidol..

[54] Patten D.A., Shetty S. (2018). More Than Just a Removal Service: Scavenger Receptors in Leukocyte Trafficking. Front. Immunol..

[55] Pfeiffer A., Böttcher A., Orsó E., Kapinsky M., Nagy P., Bodnár A., Spreitzer I., Liebisch G., Drobnik W., Gempel K. (2001). Lipopolysaccharide and ceramide docking to CD14 provokes ligand-specific receptor clustering in rafts. Eur. J. Immunol..

[56] Zanoni I., Ostuni R., Marek L.R., Barresi S., Barbalat R., Barton G.M., Granucci F., Kagan J.C. (2011). CD14 Controls the LPS-Induced Endocytosis of Toll-Like Receptor 4. Cell.

[57] Dustin M.L. (2014). The Immunological Synapse. Cancer Immunol. Res..

[58] Ao M., Wu L., Zhou X., Chen Y. (2016). Methyl-β-Cyclodextrin Impairs the Monocyte-Adhering Ability of Endothelial Cells by Down-Regulating Adhesion Molecules and Caveolae and Reorganizing the Actin Cytoskeleton. Biol. Pharm. Bull..

[59] Chen G., Zhou Y., Zhang W., Qin Y., Wei B., Sun Y., Chen Y. (2021). Methyl-β-cyclodextrin suppresses the monocyte-endothelial adhesion triggered by lipopolysaccharide (LPS) or oxidized low-density lipoprotein (oxLDL). Pharm. Biol..

[60] Del Pozo M.A. (2004). Integrin signaling and lipid rafts. Cell Cycle.

[61] Shih P.T., Elices M.J., Fang Z.T., Ugarova T.P., Strahl D., Territo M.C., Frank J.S., Kovach N.L., Cabanas C., Berliner J.A. (1999). Minimally modified low-density lipoprotein induces monocyte adhesion to endothelial connecting segment-1 by activating beta1 integrin. J. Clin. Investig..

[62] Niwa S., Totsuka T., Hayashi S. (1996). Inhibitory effect of fluvastatin, an HMG-CoA reductase inhibitor, on the expression of adhesion molecules on human monocyte cell line. Int. J. Immunopharmacol..

[63] Kawakami A., Tanaka A., Nakajima K., Shimokado K., Yoshida M. (2002). Atorvastatin Attenuates Remnant Lipoprotein-Induced Monocyte Adhesion to Vascular Endothelium under Flow Conditions. Circ. Res..

[64] Cerda A., Rodrigues A.C., Alves C., Genvigir F.D.V., Fajardo C.M., Dorea E.L., Gusukuma M.C., Pinto G.A., Hirata M.H., Hirata R.D.C. (2015). Modulation of Adhesion Molecules by Cholesterol-Lowering Therapy in Mononuclear Cells from Hypercholesterolemic Patients. Cardiovasc. Ther..

[65] Weber C., Erl W., Weber K.S.C., Weber P.C. (1999). Effects of Oxidized Low Density Lipoprotein, Lipid Mediators and Statins on Vascular Cell Interactions. Clin. Chem. Lab. Med. CCLM.

[66] Gironi B., Kahveci Z., McGill B., Lechner B.-D., Pagliara S., Metz J., Morresi A., Palombo F., Sassi P., Petrov P.G. (2020). Effect of DMSO on the Mechanical and Structural Properties of Model and Biological Membranes. Biophys. J..

[67] DeBons A.F., Fani K., AJimenez F., Maayan M.L. (1987). Inhibition of cholesterol-induced atherosclerosis in rabbits by dimethyl sulfoxide. J. Pharmacol. Exp. Ther..

[68] Kollerup M.B., Hilscher M., Zetner D., Rosenberg J. (2018). Adverse reactions of dimethyl sulfoxide in humans: A systematic review. F1000Research.

[69] Ravandi A., Babaei S., Leung R., Monge J.C., Hoppe G., Hoff H., Kamido H., Kuksis A. (2004). Phospholipids and oxophospholipids in atherosclerotic plaques at different stages of plaque development. Lipids.

[70] Yoshida H., Kisugi R. (2010). Mechanisms of LDL oxidation. Clin. Chim. Acta.

[71] Ghaffari S., Nabi F.N., Sugiyama M.G., Lee W.L. (2018). Estrogen Inhibits LDL (Low-Density Lipoprotein) Transcytosis by Human Coronary Artery Endothelial Cells via GPER (G-Protein–Coupled Estrogen Receptor) and SR-BI (Scavenger Receptor Class B Type 1). Arterioscler. Thromb. Vasc. Biol..

[72] Libby P., Pasterkamp G., Crea F., Jang I.-K. (2019). Reassessing the Mechanisms of Acute Coronary Syndromes. Circ. Res..

[73] Swystun L.L., Liaw P.C. (2016). The role of leukocytes in thrombosis. Blood.

[74] Silverstein R.L., Li W., Park Y.M., Rahaman S.O. (2010). Mechanisms of cell signaling by the scavenger receptor CD36: Implications in atherosclerosis and thrombosis. Trans. Am. Clin. Clim. Assoc..

[75] Bolick D.T., Srinivasan S., Whetzel A., Fuller L.C., Hedrick C.C. (2006). 12/15 lipoxygenase mediates monocyte adhesion to aortic endothelium in apolipoprotein E-deficient mice through activation of RhoA and NF-kappaB. Arterioscler. Thromb. Vasc. Biol..

[76] Fu G., Deng M., Neal M.D., Billiar T.R., Scott M.J. (2021). Platelet–Monocyte Aggregates: Understanding Mechanisms and Functions in Sepsis. Shock.

[77] Lewis J.C., Jones N.L., Hermanns M., Röhrig O., Klein C.L., Kirkpatrick C. (1995). Tissue Factor Expression during Coculture of Endothelial Cells and Monocytes. Exp. Mol. Pathol..

[78] Palabrica T., Lobb R., Furie B.C., Aronovitz M., Benjamin C., Hsu Y.-M., Sajer S.A., Furie B. (1992). Leukocyte accumulation promoting fibrin deposition is mediated in vivo by P-selectin on adherent platelets. Nature.

[79] Michelson A.D., Barnard M.R., Krueger L.A., Valeri C.R., Furman M.I. (2001). Circulating monocyte-platelet aggregates are a more sensitive marker of in vivo platelet activation than platelet surface P-Selectin: Studies in baboons, human coronary intervention, and human acute myocardial infarction. Circulation.

[80] Azumi H., Hirata K.-I., Ishida T., Kojima Y., Rikitake Y., Takeuchi S., Inoue N., Kawashima S., Hayashi Y., Itoh H. (2003). Immunohistochemical localization of endothelial cell-derived lipase in atherosclerotic human coronary arteries. Cardiovasc. Res..

[81] Auerbach B.J., Bisgaier C.L., Wölle J., Saxena U. (1996). Oxidation of Low Density Lipoproteins Greatly Enhances Their Association with Lipoprotein Lipase Anchored to Endothelial Cell Matrix. J. Biol. Chem..

[82] Obunike J.C., Paka S., Pillarisetti S., Goldberg I.J. (1997). Lipoprotein lipase can function as a monocyte adhesion protein. Arter. Thromb. Vasc. Biol..

[83] Hayashida K., Kume N., Minami M., Kita T. (2002). Lectin-like oxidized LDL receptor-1 (LOX-1) supports adhesion of mononuclear leukocytes and a monocyte-like cell line THP-1 cells under static and flow conditions. FEBS Lett..

[84] Duryee M.J., Clemens D.L., Opperman P.J., Thiele G.M., Duryee L.M., Garvin R.P., Anderson D.R. (2021). Malondialdehyde-Acetaldehyde Modified (MAA) Proteins Differentially Effect the Inflammatory Response in Macrophage, Endothelial Cells and Animal Models of Cardiovascular Disease. Int. J. Mol. Sci..

[85] Geloen A., Helin L., Geeraert B., Malaud E., Holvoet P., Marguerie G. (2012). CD36 Inhibitors Reduce Postprandial Hypertriglyceridemia and Protect against Diabetic Dyslipidemia and Atherosclerosis. PLoS ONE.

[86] Gargalovic P.S., Gharavi N.M., Clark M.J., Pagnon J., Yang W.-P., He A., Truong A., Baruch-Oren T., Berliner J.A., Kirchgessner T.G. (2006). The Unfolded Protein Response Is an Important Regulator of Inflammatory Genes in Endothelial Cells. Arterioscler. Thromb. Vasc. Biol..

[87] Mendel I., Feige E., Yacov N., Salem Y., Levi I., Propheta-Meiran O., Shoham A., Ishai E., George J., Harats D. (2013). VB-201, an oxidized phospholipid small molecule, inhibits CD14- and Toll-like receptor-2-dependent innate cell activation and constrains atherosclerosis. Clin. Exp. Immunol..

[88] Feige E., Yacov N., Salem Y., Levi I., Mendel I., Propheta-Meiran O., Shoham A., Hait-Darshan R., Polonsky O., George J. (2013). Inhibition of monocyte chemotaxis by VB-201, a small molecule lecinoxoid, hinders atherosclerosis development in ApoE^−/−^ mice. Atherosclerosis.

[89] Montecucco F., Lenglet S., Carbone F., Boero S., Pelli G., Burger F., Roth A., Bertolotto M., Nencioni A., Cea M. (2015). Treatment with KLEPTOSE® CRYSMEB reduces mouse atherogenesis by impacting on lipid profile and Th1 lymphocyte response. Vasc. Pharmacol..

[90] Zimmer S., Grebe A., Bakke S.S., Bode N., Halvorsen B., Ulas T., Skjelland M., De Nardo D., Labzin L.I., Kerksiek A. (2016). Cyclodextrin promotes atherosclerosis regression via macrophage reprogramming. Sci. Transl. Med..

[91] Wang H., Zhang X., Yu B., Peng X., Liu Y., Wang A., Zhao D., Pang D., Ouyang H., Tang X. (2019). Cyclodextrin Ameliorates the Progression of Atherosclerosis via Increasing High-Density Lipoprotein Cholesterol Plasma Levels and Anti-inflammatory Effects in Rabbits. J. Cardiovasc. Pharmacol..

[92] Kim H., Han J., Park J.-H. (2020). Cyclodextrin polymer improves atherosclerosis therapy and reduces ototoxicity. J. Control. Release.

[93] Kilsdonk E.P.C., Yancey P.G., Stoudt G.W., Bangerter F.W., Johnson W.J., Phillips M.C., Rothblat G.H. (1995). Cellular Cholesterol Efflux Mediated by Cyclodextrins. J. Biol. Chem..

[94] Bakke S.S., Aune M.H., Niyonzima N., Pilely K., Ryan L., Skjelland M., Garred P., Aukrust P., Halvorsen B., Latz E. (2017). Cyclodextrin Reduces Cholesterol Crystal–Induced Inflammation by Modulating Complement Activation. J. Immunol..

[95] Pilely K., Bakke S.S., Palarasah Y., Skjoedt M.-O., Bartels E.D., Espevik T., Garred P. (2019). Alpha-cyclodextrin inhibits cholesterol crystal-induced complement-mediated inflammation: A potential new compound for treatment of atherosclerosis. Atherosclerosis.

[96] Oesterle A., Laufs U., Liao J.K. (2017). Pleiotropic Effects of Statins on the Cardiovascular System. Circ. Res..

